# Transitioning from Traditional Teaching to Problem Based Learning Facilitation: A Comprehensive Tutor Training Program at Patan Academy of Health Sciences

**DOI:** 10.31729/jnma.v63i292.9247

**Published:** 2025-12-31

**Authors:** Sushant Aryal, Reshu Agrawal Sagtani, Tankeshwar Acharya, Ritu Bashyal

**Affiliations:** 1 Department of Pharmacology, Patan Academy of Health Sciences, Lagankhel, Lalitpur, Nepal; 2 Department of Community Health science, Patan Academy of Health Sciences, Lagankhel, Lalitpur, Nepal; 3 Department of Microbiology, Patan Academy of Health Sciences, Lagankhel, Lalitpur, Nepal; 4 Department of Biochemistry, Patan Academy of Health Sciences, Lagankhel, Lalitpur, Nepal

**Keywords:** *facilitator*, *problem-based learning*, *training*, *tutor*

## Abstract

Tutors play a key role in the success of a Problem-Based Learning program. Effective training is essential to prepare them for this role. To support new faculty, Patan Academy of Health Sciences developed a comprehensive four-phase tutor training program, which includes orientation, tutor shadowing, supervised tutoring, and supervised student assessment. Training is delivered by Patan Academy of Health Sciences faculty with formal Problem-Based Learning training and extensive facilitation experience. The program combines theoretical knowledge with hands-on practice to help tutors develop the skills needed to facilitate Problem-Based Learning effectively. Trainees are evaluated by trainer and awarded certification upon meeting competency standards. This program has enhanced the quality and success of Problem-Based Learning at Patan Academy of Health Sciences. Its effectiveness is regularly assessed using participant feedback and student assessments of tutors to ensure continuous improvement.

## INTRODUCTION

Problem-based learning (PBL) is a student-centered educational method where learning is initiated by presenting students with ill-structured real-world problem. This method promotes active learning and help indents develop essential generic skills including problem solving, critical thinking, communication, self directed learning and team work.^1^ In PBL, the tutor’s role shifts from a traditional teacher to a facilitator which requires a distinct set of skills. Tutors need a thorough understanding of the PBL process and must have skill to facilitate group discussions, manage group dynamics, assess students and give them feedback.^2-5^ The success of PBL program depends significantly on the competence of PBL tutors. However, shifting to the role of PBL tutor involves a major change and a significant challenge.^6,7^ Therefore, structured tutor training, including orientation and tutor shadowing are essential for developing PBL tutors with the necessary skills for the effective implementation of PBL.^8-10^ This paper describes the design, implementation, and initial outcomes of a comprehensive PBL tutor training program developed at Patan Academy of Health Sciences.

## NEED FOR STANDARDIZED TUTOR TRAINING AT PATAN ACADEMY OF HEALTH SCIENCES

Over the past decade of implementing PBL at Patan Academy of Health Sciences (PAHS), we have observed that new faculty find it challenging to shift from traditional teaching roles to facilitating PBL session. Faculty members joining PAHS come from diverse academic backgrounds and institutions with unique pedagogical methods. While this diversity helps in enhancing the educational experience at the institution, it also reinforces the need for a standardized training program to ensure all tutors are well-prepared to succeed in their roles.

In earlier practice, newly appointed teaching faculty at PAHS underwent a conventional two-day training that included the theoretical foundations of PBL and observation of mock sessions. However, this approach had several limitations. Training sessions were often delayed because a full group of participants had to be assembled. Additionally, there was no opportunity to observe or engage in actual PBL sessions, making it difficult for new tutors to develop the skills and confidence needed to effectively facilitate student learning. Furthermore, no formal assessment mechanism was in place to evaluate tutor competency or readiness prior to their involvement in real PBL sessions.

A comprehensive training program was introduced to address these gaps with the objectives to equip faculty with in-depth understanding of the principles, processes, and assessment methods associated with PBL as well as to provide hands-on experience to ensure practical application.

## DETAILS OF THE COMPREHENSIVE PBL TUTOR TRAINING PROGRAM

### Training design

The Comprehensive PBL Training Program was developed by the PBL Committee and endorsed by the Health Professional Education Unit at PAHS. It is delivered by experienced PAHS faculties who have received formal PBL training and have more than ten years of facilitation experience. All newly appointed faculty members undergo this training before being assigned as independent tutors. The program is organized into four structured phase, beginning with an orientation and followed by three phases integrated with the ongoing MBBS tutorial sessions. Each phase focuses on specific learning outcomes and skill development of the new tutor.

### Phase 1: Interactive Orientation

The training begins with a two-day interactive orientation to introduce the theoretical foundations of PBL. One week before the orientation, trainees are enrolled in Moodle learning platform of PAHS. All relevant training materials including, reading resources, session outlines and references material are uploaded to facilitate their preparation.

During the orientation, participants engage in interactive sessions with PBL committee members, encouraging open discussion and active participation. Topics covered includes history and evolution of PBL, its core principles,rationale for its use in medical education, step-by-step PBL process, importance of feedback and feedback techniques, role of small group dynamics in PBL and strategies to manage it, responsibilities of tutors and students, common challenges encountered during PBL and how to address them, and strategies for designing PBL cases.

### Phase 2: Tutor shadowing

This phase lasts for two weeks during which, trainee is assigned to a supervising trainer. Trainee observes the trainer facilitating PBL and wrap up sessions. The main objective is to provide the opportunity to observe the complete process of PBL. The observation is followed by restective discussion between supervising trainer and trainee at the end of each session to address trainee’s queries and clarify unfamiliar concepts.

### Phase 3: Supervised Tutoring

This phase is of two weeks. In this phase, trainee facilitates PBL sessions under the supervision of a trainer. The primary objective of this phase is to enable trainee to conduct PBL sessions independently. After each session, they receive constructive feedback from trainer to help them improve their performance.

### Phase 4: Supervised PBL process assessment of students

The objective of this phase is to enable trainee on independent use of the Tutor Assessment of Student (TAS) tool and provide individual feedback to students based on their performance. During this phase, trainees complete the TAS tool and deliver individualized feedback to students under the supervision of a trainer.

### Certification

Upon completing the training, trainee’s competency is evaluated by supervising trainer using the Tutor Assessment of Co-Tutor tool. Those not meeting expectation, based on the feedback received from the trainer, additional week/s of supervised training is made to optimize trainee’s tutoring skill.

## CONCLUSION

The conventional PBL tutoring program had limitations, including delays in training, no real time observation for PBL session, limited hands-on practice, and insufficient feedback for new tutors. The comprehensive PBL training program addresses these gaps by providing structured and practical training. Furthermore, because this training program is integrated with the on-going MBBS PBL tutorial session, it can be delivered in a timely manner to newly appointed faculty, without the need to assemble a large group of trainees as required in the conventional training. This training has been a valuable step in preparing faculty members to competently facilitate PBL sessions and has contributed to the overall success of the PBL program.

To continuously improve this training program, participants’ feedback and student assessments of tutors are used. This helps to assess the effectiveness of the program.
